# Secular trends in family dinner frequency among adolescents

**DOI:** 10.1186/s13104-016-1856-2

**Published:** 2016-01-22

**Authors:** Kathryn Walton, Ken P. Kleinman, Sheryl L. Rifas-Shiman, Nicholas J. Horton, Matthew W. Gillman, Alison E. Field, S. Bryn Austin, Dianne Neumark-Sztainer, Jess Haines

**Affiliations:** Department of Family Relations and Applied Nutrition, University of Guelph, 50 Stone Road East, Guelph, ON N1G 2W1 Canada; Obesity Prevention Program, Department of Population Medicine, Harvard Medical School and Harvard Pilgrim Health Care Institute, Boston, USA; Department of Mathematics and Statistics, Amherst College, Amherst, USA; Department of Nutrition, Harvard T.H. Chan School of Public Health, Boston, USA; Division of Adolescent and Young Adult Medicine, Boston Children’s Hospital, Boston, USA; Channing Division of Network Medicine, Brigham and Women’s Hospital and Harvard Medical School, Boston, USA; Department of Social and Behavioral Sciences, Harvard T.H. Chan School of Public Health, Boston, USA; Department of Epidemiology, Harvard T.H. Chan School of Public Health, Boston, USA; Division of Epidemiology and Community Health, School of Public Health, University of Minnesota, Minneapolis, USA

**Keywords:** Family meals, Longitudinal studies, Secular trends, Adolescents

## Abstract

**Background:**

Eating meals, particularly dinner, with family members has been found to be associated with improved dietary intake, lower prevalence of disordered eating behaviors, lower levels of substance abuse, and improved academic outcomes among adolescents. Limited research has examined how the frequency of family meals has changed over time. The objective of this study was to examine secular trends in family dinner frequency over a 12-year period using a large, nation-wide sample of adolescents.

**Methods:**

Using data from two cohorts of the Growing up Today study (GUTS; n = 18,075 observations for 14,79,714 and 15 year olds), we compared family dinner frequency among 14–15-year-olds in 1996 (GUTS1) through 2008 (GUTS2) and rate of change in family dinner frequency from 1996 to 1998 (GUTS1) and 2004–2008 (GUTS2). We fit logistic models using generalized estimating equations with independence working correlation and empirical variance to account for correlation within individual and between siblings.

**Results:**

From 1996 to 2008, the number of family dinners per week among males decreased from 5.3 to 4.6 (p = 0.04) and among females from 5.0 to 4.4 (p = 0.03). We found that the rate of decline in frequency of family meals was consistent in GUTS1 (1996–1998) and GUTS2 (2004–2008) among both males and females.

**Conclusions:**

From 1996 to 2008, frequency of family dinners decreased among adolescents. Future research should explore reasons for this decline as well as strategies to increase family meals among adolescents.

## Background

Eating dinner with family members is associated with improved dietary intake [1], lower prevalence of disordered eating behaviors [2–5], lower levels of substance abuse [3, 6], and improved academic outcomes among adolescents [6]. Given the potential importance that family meals play in adolescents’ physical and mental well-being, understanding whether family meal frequency has changed over time is an important question that few studies have explored.

The two studies that have examined secular trends in family meal frequency among American youth found conflicting results. The first study, a non-peer reviewed white paper, examined a series of cross-sectional studies of approximately 1000 American youth and found that the percentage of youth reporting five or more family meals per week remained relatively stable from 1999 to 2011 [7]. Neumark-Sztainer and colleagues [8] explored secular trends in family meals among a sample of 3000 Minnesotan youth and found that, among their overall sample, the percentage of youth reporting eating meals with their family five or more times per week decreased from 1999 to 2010. However, this trend differed by socio-economic status; the frequency of family meals decreased among adolescents from families of low socio-economic status and increased among adolescents from families of high socio-economic status [8]. Given that there has only been a single peer-reviewed study examining secular trends in adolescent family meal participation in a sample limited to a single geographic region, it is unclear whether or not family meal frequency has changed in the United States over the recent decade.

The Growing Up Today Study involves two on-going, nation-wide cohort studies; GUTS1 assessed family dinner frequency among a prospective cohort of adolescents in 1996, 1997 and 1998 and GUTS2 assessed family dinner frequency among a prospective cohort of similarly aged adolescents in 2004, 2006 and 2008. Because these cohorts assessed family dinner frequency across multiple years within two different decades, these data provide the unique opportunity to examine how the frequency of family dinners has changed over a 12-year period among adolescents (i.e., secular trends) and to compare the rate of change in family dinner frequency across these decades. The primary aim of this study was to examine secular trends in family dinner frequency from 1996 to 2008 in a large, nation-wide cohort of adolescents. The secondary aim was to examine whether the rate of change in family dinner frequency differed between 1996–1998 and 2004–2008. Exploring secular trends in family meal frequency in a large, nation-wide sample may provide insight into whether family meal norms and practices have changed over a 12-year period of substantial economic upheaval [9].

## Methods

### Study design and population

We examined trends in family dinner participation among participants of the Growing Up Today Study (GUTS). GUTS consists of two on-going cohort studies of offspring of nurses (participants of the Nurses Health Study II (NHS II) [10]). The first cohort, GUTS1, was established in 1996 and the second cohort, GUTS2, was established in 2004. For the GUTS1 cohort, we contacted mothers in the NHS II who we identified to have an adolescent between the ages of 9–14 years through mailed letters describing the purpose of GUTS and requesting permission to contact their children. In 1996, we mailed questionnaires to 13,261 females and 13,504 males whose mothers had granted consent; 9039 (68 % response rate) females and 7843 (58 % response rate) males returned completed questionnaires, thereby assenting to participate in GUTS1. Follow-up questionnaires were mailed annually in 1997 and 1998. A similar process was followed for recruitment in the GUTS2 cohort; 20,700 women in NHS II who had children aged 9–17 years were contacted and, in 2004, we mailed questionnaires to 8826 females and 8454 males whose mothers granted consent to contact their child. A total of 6002 (68 % response rate) females and 4918 (58 % response rate) males returned completed questionnaires, thereby assenting to participate in GUTS2. Follow-up questionnaires (on-line and mailed paper copies) were sent biannually in 2006 and 2008. This study was approved by the Human Subjects Committee at the Brigham and Women’s Hospital.

For this study, we restricted analysis to participants who were aged 14 or 15 years during the years when frequency of family dinners was assessed (1996–1998 for GUTS1, n = 11,131 observations for 7779 participants; and 2004–2008 for GUTS2, n = 8071 observations for 7987). This age range was selected as it was represented in each year that family dinner was assessed. We excluded observations with missing family dinner data (n = 1041 observations in GUTS1 and 86 observations in GUTS2) from these analyses, resulting in an analytic sample of 10,090 observations for 6889 participants in GUTS1 and 7985 observations for 7908 participants in GUTS2. In the analytic sample, 78 % had one family dinner observation and 22 % had more than one observation and 20 % had at least one sibling in the cohort.

### Measures

#### Family dinner frequency

Family dinner frequency was measured in GUTS1 in 1996, 1997 and 1998 and in GUTS2 in 2004, 2006 and 2008 using the question: “How often do you sit down with other members of your family to eat dinner or supper?” In GUTS1, response categories were, “never,” “some days,” “most days,” and “every day.” In GUTS2, the responses were “never/almost never,” “1–2 times/week,” “3–4 times/week,” and “5 or more times/week.” For GUTS1, we coded the response option “never” as 0 times/week, “some days” as 3 times/week, “most days” as 5 times/week, and “everyday” as 7 times/week. For GUTS2, we coded the response option “never/almost never” as 0.07 times/week, “1–2 times/week” as 1.5 times/week, “3–4 times/week” as 3.5 times/week, and “5 or more times per week” as 6 times/week. To explore whether using different values to represent the response options in GUTS1 would influence our results, we also explored alternative values (never = 0, some days = 1, most days = 3.6, and every day = 7); analyses using these alternative values produced similar results (results not shown).

#### Household socioeconomic status

As part of the NHS II surveys, mothers reported their annual household income in 2001 and the education level of their partners in 1999.

#### Analysis

We stratified all analyses by sex as previous research found frequency of family dinners to differ among males and females [8].

To examine secular trends in family dinner among 14- and 15-year-olds in 1996 through 2008, we first calculated mean and standard deviation of the number of family dinners for each year. Then, to model family dinner frequency, we fit logistic models using generalized estimating equations with independence working correlation and empirical variance to account for correlation within individual and between siblings [11, 12]. The outcome was the number of family dinners per week, treated as a binomial random variable with n = 7 trials. We included a covariate indicating GUTS1 (1996–1998 vs. GUTS2 2004–2008), time (1996 coded as 0 to 2008 coded as 12), and the interaction of GUTS1 by time (to test linear trends for GUTS1 and GUTS2). The interaction term was not significant, indicating that the rate of change in family dinner frequency was similar across GUTS1 and GUTS2, so we removed it from the models. We fit models that additionally adjusted for household income and maternal partner’s education level; the effect estimate remained virtually unchanged (results not shown), so we report only estimates from the more parsimonious, unadjusted model. We conducted our analyses using SAS (version 9.3, SAS Institute Inc., Cary NC, USA).

We also explored the 12-year secular trends and rate of change stratified by annual household income (<$75,000/year and ≥$75,000/year) and maternal partner’s education level (less than a college education and a college education or higher) [8]. Results from these stratified models showed that the decrease in family meals and rate of change in family meals were similar across income and maternal partner education levels (results not shown).

## Results

Between the GUTS1 and GUTS2 cohorts, 14,797 participants contributed to an analytic sample of 18,075 observations of 14 and 15 year olds. Of these observations, the majority of adolescents identified as being white (93 % in both GUTS1 and GUTS2). Overall, 43 % of observations were for males. Observations were split equally between 14 (55 %) and 15 (45 %) year olds in our analytical sample. The majority of adolescents were from higher socio-economic homes, with 62 % of their mothers reporting household incomes ≥ $75 000/year in 2001 and 66 % of their mothers reporting their partner had at least a college education in 1999.

**Fig. 1:**
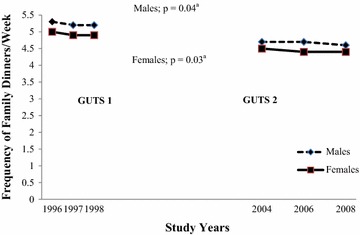
Secular Trends in Family Dinner Frequency between 1996 and 2008 among 14- and 15-year-old adolescents. ^a^p-values represent statistically significant decline in family dinner frequency over the study years by gender based on logistic regression models

From 1996 to 2008 the mean (SD) number of family dinners per week among males decreased from 5.3 (1.6) to 4.6 (1.8) (p = 0.04; Fig. 1). The odds of having a family dinner decreased each year by −0.02 (95 % CI −0.05, −0.001) among males.

Among females, the mean (SD) number of family dinners per week decreased from 5.0 (1.7) to 4.4 (1.8) dinners/week from 1996 to 2008 (p = 0.03; Fig. 1). The odds of having a family dinner decreased each year by −0.02 (95 % CI −0.04, −0.002).

We found no significant difference in the rate of decline in frequency of family dinners between GUTS1 (1996–1998) and GUTS2 (2004–2008) among males or females.

## Discussion

Our findings suggest that over the 12-year span of 1996–2008, the frequency of family dinners among our sample of offspring of nurses decreased and that the rate of decline was consistent across time. Our finding that the frequency of family dinners is decreasing is of concern, given that family meals have been associated with lower substance abuse [3, 6], improved dietary intakes [1, 13], and improved adolescent well-being [7]. Adolescence is a time of rapid growth; therefore nutritional adequacy is of paramount importance during this life stage. Family dinners have been associated with improved dietary intakes [13, 14] with participation in family dinners being inversely associated with eating prepackaged dinners, known to be lower in nutrient value [15]. Our finding that girls participate in fewer family dinners than boys is also of concern considering previous research from longitudinal studies suggests that shared family dinners have a protective factor for disordered eating patterns among girls, but not among boys [5, 16]. While autonomy from parents increases during adolescence, literature suggests that the family environment, specifically family meals are associated with positive outcomes related adolescent behaviour and development [10].

Our findings differ from the previous peer-reviewed study by Neumark-Sztainer et al. [8], who found that, among adolescents from higher socio-economic homes, (defined by adolescent report of parent have at least a college education), family meals increased from 4.2 meals per week in 1999 to 4.5 meals per week 2010 (p = 0.039). Although our sample included a majority of adolescents from higher SES homes, family dinner frequency declined in our sample over a similar time period. The geographic diversity of our sample may explain the difference in results. While participants in the Neumark-Sztainer study are socio-economically diverse, they included adolescents from only one metropolitan area of Minnesota, Minneapolis/St. Paul and thus, their results may not be generalizable to populations from other regions of the United States [8]. It is also possible that our results differ because our participants are offspring of nurses. The stressors associated with the nursing profession (vs. other professions requiring a college education), including irregular working hours and evening shifts, may be different than many other professions that require having a college education. However, our finding that family dinner participation is decreasing among adolescents with parents who are highly educated suggests that future interventions should target families with parents who have high as well as low education levels. Our results also suggest that future etiologic research should explore how other aspects of the family environment, such as parental work schedules and stressors, may influence family dinner participation.

The rate of decline in the frequency of family dinners was consistent in GUTS1 (1996–1998) and GUTS2 (2004–2008) among both males and females. The U.S. experienced an economic recession during the years the GUTS2 data was collected [9]. According to the U.S. Department of Agriculture, average household spending on food prepared outside the home decreased during that time by 12.9 % between the years of 2006 and 2009 [17]. Based on this information, we may have expected the decline in family dinner participation in our sample to be more precipitous in the earlier years (GUTS1) versus the later years (GUTS2) [9]. It is possible that our sample of families were not sufficiently financially impacted by the recession to change food purchasing practices. It is also possible that fewer meals consumed away from home did not result in families being more likely to share a meal together. Future research should explore the reasons why families are having fewer meals together and effective ways to translate research findings on the impact family meals may have on adolescent wellbeing. This information could help inform policy or family-level interventions to support regular family meals.

When interpreting our findings, limitations should be noted. First, we presented data only from 14- and 15-year-olds; results may not be indicative of family dinner trends among younger or older adolescents. Research indicates that increased independence from parents increases linearly from age 9 to approximately age 13 years and thus we may have missed capturing a period in earlier adolescence where the frequency of family dinner may have been changing at a different rate [18]. Second, caution must be used when generalizing the results to other populations; the majority of participants identified as being white. Furthermore, participants were children of nurses; thus, our participants come from homes with highly educated parents that may place a higher importance on a healthy lifestyle as compared to the general population. We only explored family dinners. It is possible that families eat other meals together that are not captured in this study. However, findings from [19] suggest that there is no statistically significant differences in studies reporting a protective factor for family meals when measuring family meals in general versus dinner only, suggesting that participants may subjectively interpret the family meal to mean dinner. Finally, response options to the frequency of family meals differed between the GUTS1 and GUTS2 cohorts. While sensitivity analyses using alternative responses did not impact results, these varying response options across the cohorts could have potentially misclassified participants’ family dinner participation. Despite these limitations, our study is an important addition to the existing literature as it provides information on secular trends on the frequency of family dinner intake over a 12-year time period among a large, nation-wide sample of adolescents. Additionally, during this 12-year period, we compared rate of change in family dinner frequency in two different decades as well as concentrated years within these decades. This provides a more comprehensive understanding of adolescent family dinner participation during our study period. Finally, our sample size in this study was sufficiently large to assess the associations between family dinner frequency separately for males and females at each time point.

## Conclusions

From 1996–2008, frequency of family dinners decreased among a nation-wide sample of adolescents aged 14–15 years who are offspring of nurses. These results add to the small body of peer-reviewed research exploring secular trends in family meals and suggest the need for future research to explore the reasons why families are participating in fewer dinners together and explore strategies to increase frequency of family meals among families with adolescents. Future interventions may need to target specific factors of the family environment such as the time pressures and stressors associated with parent profession.
